# Compression syndromes of the popliteal neurovascular due to Baker cyst: A case report

**DOI:** 10.1016/j.ijscr.2023.108013

**Published:** 2023-03-21

**Authors:** Erica Kholinne, Endrotomo Sumargono, Dyonesia Ary Harjanti, Ira Juliet Anestessia

**Affiliations:** a Faculty of Medicine, Universitas Trisakti, Jakarta, Indonesia; b Department of Orthopedic Surgery, St. Carolus Hospital, Jakarta, Indonesia; c Faculty of Medicine, University of Atma Jaya, Jakarta, Indonesia

**Keywords:** Case report, Baker cyst, Meniscal cyst, Popliteal cyst, Neurovascular compression, Excision

## Abstract

**Introduction and importance:**

Baker's cyst, or popliteal cyst, is typically arising in the popliteal fossa located between the semi-membranous tendon and the medial head of the gastrocnemius. Asymptomatic Baker's cyst does not require treatment. Surgical treatment may be considered after the failure of conservative measures when the cyst causes persistent pain and rarely compresses the adjacent neurovascular structure.

**Case presentation:**

We report an unusual presentation of Baker's cyst in a 43-year-old patient who complained of knee pain and after several months of conservative treatment. Following a physical and radiological examination, a Baker cyst was confirmed that compresses the tibial nerve. A surgical decompression and excision of the cyst was performed using the posterior approach to allow complete removal of the cyst's stalk and wall. Histopathological report confirms dense collagen without true epithelial lining. The patient was asymptomatic at 6 months of follow-up.

**Clinical discussion:**

It is important to recognize that a large Baker's cyst can be symptomatic and present as a compression syndrome of the adjacent neurovascular structure. The current case report described an early surgical management to treat a tibial nerve compression syndrome caused by a Baker cyst. A surgical decompression through a posterior approach may facilitate complete removal of the cyst wall.

**Conclusions:**

Baker's cyst is a common knee pathology that could rarely compress the adjacent neurovascular structures. The surgical decompression through a posterior approach results in favorable outcomes in symptomatic patients with failed conservative measures.

## Highlights

• Baker’s cyst could be presented as a compression syndrome (of the tibial nerve)
• Failed conservative treatment with deterioration of lower limb function necessitate surgical decompression
• Surgical decompression allow complete removal of the ventral wall and stalk of the cyst, hence can be considered safe, and reproducible procedure

## 1 Introduction

Baker's cyst, or popliteal cyst, is usually arising from the popliteal fossa located between the semimembranosus tendon and the medial head of the gastrocnemius [1]. Many knee pathologies have been associated with Baker's cyst, such as meniscal injury, effusion, chondral lesions, anterior cruciate ligament tears, degenerative and inflammatory osteoarthritis [2]. Of all associated conditions, the cyst is commonly related to degenerative osteoarthritis and meniscal injury [2]. William Morant Baker described the cyst in 1877, which later had his name honoured for the eponym despite that several surgeons described this entity before him [2], [3]. Occasionally, communication between the cyst and the cavity of the knee joint prevents the cyst drain its fluid material into the joint. The incidence of Baker's cysts may vary by the population and diagnostic imaging used [4], [5], [6], [7]. Baker's cysts are mostly asymptomatic, however, it can present as a source of knee pain regardless of surgical treatment of concomitant intra-articular pathology. The initial management for a symptomatic baker's cyst is conservative measures for at least 6 weeks. Conservative treatment is not preferable if there is neurovascular compression resulting in a significantly larger cyst [8]. A compression of the adjacent neurovascular bundle could cause symptoms such as thrombosis of the vascular bundle (pseudothrombophlebitic syndrome and arterial compression with claudication) or compression neuropathy (tibial or common peroneal neuropathy) [9]. The current study has been reported in accordance with the SCARE 2020 standards [10].

## 2 Presentation of case

We report a case of Baker's cyst in a middle-aged housewife whom we saw at the outpatient clinic of St. Carolus Hospital, Jakarta, Indonesia. She suffered from a trivial trauma about 6 months before admission. She lost her balance because her left foot was caught by an uneven pedestrian. She complained of mild pain at the back of the knee. She also experienced discomfort from the knee to the calf region while doing daily activities. There was occasional numbness of the sole, but no weakness of the ankle and toe was found. She did not complain of mechanical symptoms (such as locking and clicking) and swelling.

**Fig. 1:**
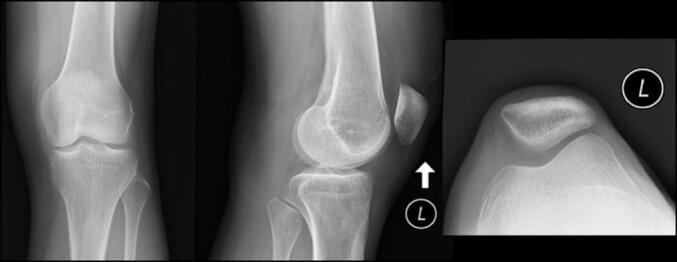
The plain radiograph of the left knee joint.

Physical examination revealed a popliteal mass aggravated by knee extension. The cyst was firm in full extension and tender when the knee is flexed. There was no joint line tenderness. Palpation of the mass was with discomfort but no pain. The circumferential measurement of both calves is the same. The range of motion is unremarkable. The integrity of the menisci was tested with Appley's and McMurray's tests which demonstrate a negative finding. The Homan sign which is a finding for pseudo-thrombophlebitis sign was absent. There was a hypesthesia of the posterolateral side of the calf and plantar foot. Motor functions remain normal. The pulsation of dorsalis pedis and the posterior tibial arteries were normal. The plain standing radiograph of the knee showed a slight narrowing of the tibiofemoral joint and a shadow of a soft tissue mass on the posterior side of the knee (Fig. 1).

**Fig. 2:**
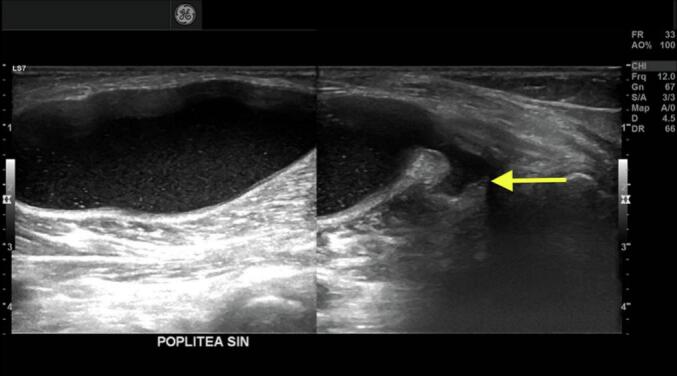
The ultrasonogram of the popliteal mass confirmed a cyst.

Ultrasound imaging of the knee revealed a large popliteal cyst containing fluid from the medial head of the gastrocnemius without any haematoma. The cyst was 8.2 × 7.4 cm in size, extending posterolaterally compressing the neurovascular bundle (Fig. 2). The laboratory work of the D-dimer was within the normal range. A duplex ultrasound was not performed due to the absence of subjective signs of deep vein thrombosis such as edema, hyperaemia, and skin discolouration of the affected extremity.

The patient was diagnosed with a Baker's cyst with compression of the tibial nerve. The patient was conservatively managed for 8 weeks with rest, a short course of non-steroidal anti-inflammatory therapy, and physiotherapy. Despite conservative measures, the patient is still symptomatic.

**Fig. 3:**
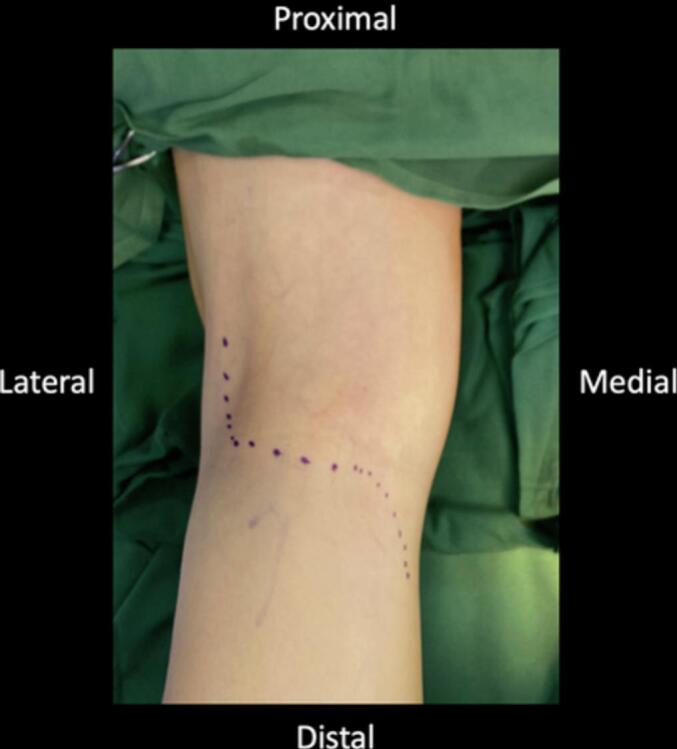
A Lazy-S (curve-linear) incision was made and centered at the cyst.

**Fig. 4:**
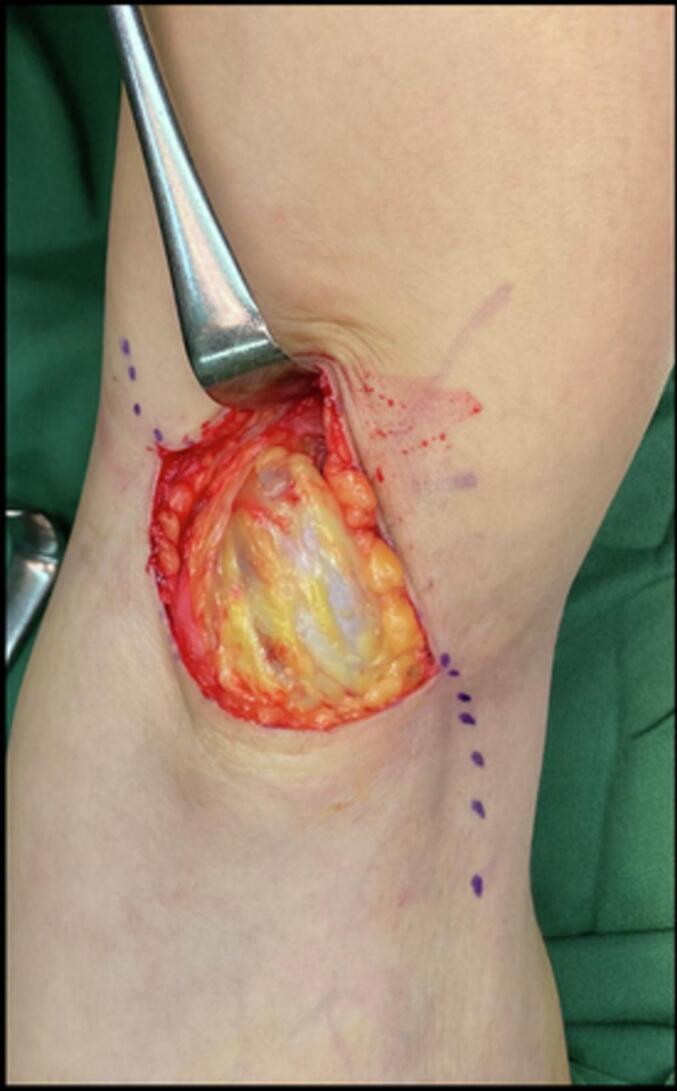
The posterior fascia was incised according to the skin incision which was later repaired at the end of the procedure.

**Fig. 5:**
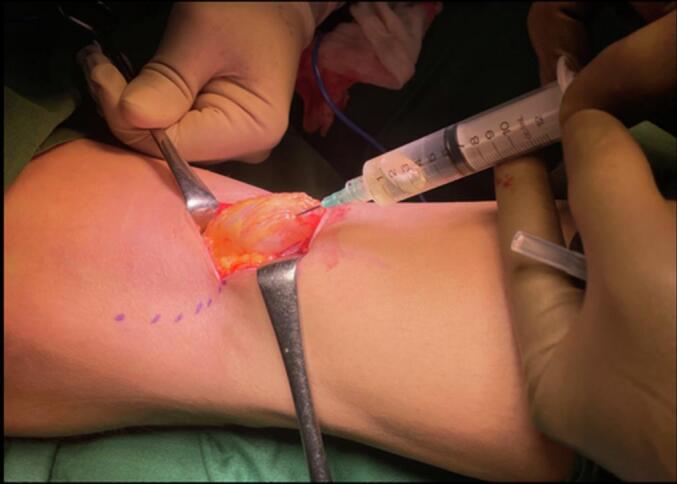
Cyst was decompressed to maximize surgical field visibility.

**Fig. 6:**
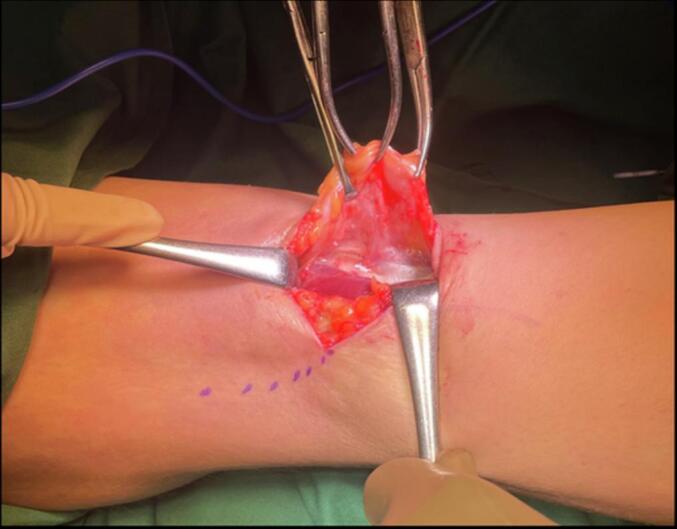
The intact cyst wall was secured with clamps.

**Fig. 7:**
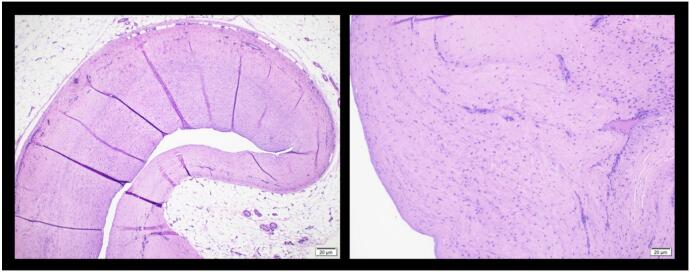
The histology report showed a dense collagenous wall with no true epithelial lining.

Early surgical measures were performed for the patient due to failed conservative treatment. The patient underwent excision of the Baker's cyst with a posterior approach. The patient was prepared in a prone position after regional anaesthesia. The operated limb was exsanguinated, and the tourniquet was inflated. The surgical incision was designed as a lazy curvilinear incision centered on the Baker cyst. A 5 cm incision was made and proceeded to the subcutaneous tissue (Fig. 3). Meticulous dissection with Metzenbaum scissors was performed carefully to respect the neurovascular bundle on the lateral side. The posterior fascia of the knee was identified and incised according to the skin incision (Fig. 4). Blunt dissection was carried out to isolate the cyst wall. The cyst was found between the semimembranosus and the medial head of the gastrocnemius. At this stage, the visibility of the surgical view was limited by the massive size of the cyst. For this reason, the cyst was aspirated with a syringe (Fig. 5). A 20 cm^3^ viscous yellowish liquid was decompressed from the cyst and the fluid was sent for analysis. The intact cyst wall was secured with clamps (Fig. 6). Blunt and sharp dissections continue until its proximolateral border which is close to the neurovascular bundle. Elevation of the cyst revealed that the cyst stalk is located in the posteromedial capsule of the knee joint. The cyst is completely excised while respecting the integrity of the posterior joint capsule. The cyst was sent to pathology for biopsy. The histological study showed a dense collagenous wall with no true epithelial lining (Fig. 7). Popliteal fossa or floor was evaluated for remaining tissue using a generous retraction of the medial head, gastrocnemius, and semimembranosus. Bleeding was meticulously controlled after tourniquet deflation. The posterior joint fascia was repaired with the absorbable suture No. 1 (Vicryl) suture (Ethicon, Somerrville, NJ). The skin was closed with a subcutaneous continuous suture.

The patient was discharged the following day with an axillary crutch (partial weight bearing).

The patient was allowed to fully weight bear 2 weeks after the operation. She was asymptomatic after 3 and 6 months of follow-up. No follow-up sonogram was performed after surgery.

## 3 Discussion

The exact cause of a popliteal cyst remains an enigma. However, it is generally accepted that the popliteal cyst is highly associated with intra-articular pathology, such as internal derangement of the knee. When the etiology of the cyst is associated with intra-articular pathology, such as meniscal injury, a surgeon should address the background disease to prevent cyst recurrence, which is usually performed with arthroscopic surgery. The patient presented in the current report was a popliteal cyst aggravated by recent trauma. The cyst was located in the posteromedial popliteal fossa due to the lack of structural resistance in this anatomic area of the joint capsule [11]. The patient also complained of occasional numbness in the sole that may be caused by compression of the tibial nerve [12]. The compression of the vascular structure is less frequent because it is located deeper. A d-dimer test was performed on the patient because the cyst occupying the lesion may mimic the symptoms of deep vein thrombosis and rule out cyst rupture. If the cyst ruptures, unnecessary anticoagulant therapy will be harmful to the patient because it can cause compartment syndrome [4].

In 2007, Ji JH et al. reported patients with calf atrophy and foot drop that were caused by Baker's cyst located between the medial head of the gastrocnemius muscle and the semi-membranosus tendon. The same case had been reported earlier in 1994 by DiRisio et al. that tibial nerve branch compression resulted in leg pain and gastrocnemius atrophy due to a cyst in the anterior of the medial head gastrocnemius dan posteromedial to the joint capsule. This is a common manifestation of neurovascular compression because the tibial nerve is the most superficial and medial structure in the popliteal fossa.

In 1970, Rauschning and Lindgren described the posterior approach for popliteal cyst excision in 41 patients [1]. The open surgical technique was not favored since the advent of knee arthroscopy because it was traditionally associated with high rates of recurrence due to inadequate management of the underlying intra-articular etiology. However, the reported patient had no mechanical symptoms of the affected knee until recent trauma. The open posterior popliteal cyst excision was chosen to facilitate satisfactory exposure of the posterior stalk or valve and adequate closure of the posterior capsule, which are relevant to prevent cyst recurrence. Furthermore, a complete cyst excision is necessary due to the compression syndrome seen in the reported case.

The open posterior approach, traditionally, has been associated with high rates of recurrence due to inadequate excision or management of the underlying intra-articular pathology and care must be taken to avoid neurovascular structure. Snir et al. [13] described that the open posterior approach is a safe, effective, and straightforward treatment option for patients with recalcitrant or idiopathic popliteal cysts because the posterior technique allows better visualization and complete removal of the cysts while minimizing the risk complications and soft tissue damage.

## 4 Conclusions

Baker's cyst, or popliteal cyst, is typically arising in the popliteal fossa located between the semi-membranous tendon and the medial head of the gastrocnemius. We reported a 43-year-old female who underwent an open posterior excision for a Baker cyst that was caused by compression of the tibial nerve. Posterior excision is a safe and straightforward treatment option for patients. Intraoperative decompression can facilitate complete removal of the ventral cyst wall.

## Patient consent

Written informed consent was obtained from the patient for publication of this case report and accompanying images. A copy of the written consent is available for review by the Editor-in-Chief of this journal on request.

## Ethical approval

Ethical approval is exempt/waived at our institution.

## Funding

Faculty of Medicine, Universitas Trisakti, Jakarta, Indonesia

## Guarantor

Erica Kholinne.

## Research registration number

N/A.

## CRediT authorship contribution statement

Erica Kholinne, Endrotomo Sumargono: Conceptualization, Methodology; Ira Juliet Anestessia, Erica Kholinne, Dyonesia Ari Harjanti: Data curation, Writing - Original draft preparation; Dyonesia Ari Harjanti, Ira Juliet Anestessia: Visualization, Investigation; Endrotomo Sumargono: Supervision; Erica Kholinne: Validation; Erica Kholinne: Writing - Reviewing and editing.

## Declaration of competing interest

None declared.
